# Reemerging Leptospirosis, California

**DOI:** 10.3201/eid1003.030431

**Published:** 2004-03

**Authors:** Elissa Meites, Michele T. Jay, Stanley Deresinski, Wun-Ju Shieh, Sherif R. Zaki, Lucy Tompkins, D. Scott Smith

**Affiliations:** * Stanford University School of Medicine, Stanford, California, USA; † California Department of Health Services, Sacramento, California, USA; ‡ Santa Clara Valley Medical Center, San Jose, California, USA; § Centers for Disease Control and Prevention, Atlanta, Georgia, USA; ¶ Kaiser Permanente Medical Group, Redwood City, California, USA

**Keywords:** Leptospirosis, Disease Outbreaks, Communicable Diseases, Emerging, California, Fresh Water, Recreation, Fever of Unknown Origin, Environmental Medicine

## Abstract

Leptospirosis is a reemerging infectious disease in California. Leptospirosis is the most widespread zoonosis throughout the world, though it is infrequently diagnosed in the continental United States. From 1982 to 2001, most reported California cases occurred in previously healthy young adult white men after recreational exposures to contaminated freshwater. We report five recent cases of human leptospirosis acquired in California, including the first documented common-source outbreak of human leptospirosis acquired in this state, and describe the subsequent environmental investigation. Salient features in the California cases include high fever with uniform renal impairment and mild hepatitis. Because leptospirosis can progress rapidly if untreated, this reemerging infection deserves consideration in febrile patients with a history of recreational freshwater exposure, even in states with a low reported incidence of infection.

Leptospirosis was distinguished and described in the early 18th century by Adolf Weil and several other scientists, although several references to epidemic jaundice and bilious typhoid likely related to leptospirosis appeared in many ancient cultures. The syndrome’s many colorful names include “rice-harvest jaundice” in China, “autumn fever” in Japan, “swineherd’s disease” in Europe, and “sewerman’s flu” in the United States (*1*). Leptospirosis is a reemerging infectious disease, involving both rural and urban cases (*2*–*6*).

Leptospira are transmitted in the urine of chronically infected carrier animals. Numerous serovars of pathogenic *Leptospira interrogans* are known to infect humans. Rats are universal reservoirs for this spirochetal zoonosis, although farm animals and livestock can also harbor the infection. In disease-endemic areas, cattle are vaccinated against leptospirosis. Infection occurs when spirochetes in contaminated water or soil enter microabrasions on the skin or intact mucous membranes (*1*–*3*,*7*). The incubation period is approximately 10 days (range 4–19 days) (*8*). In temperate climates, peak incidence is during the summer, when leptospires survive longer in the environment (*3*), and water exposures may be more common.

Leptospirosis is an acute febrile illness with nonspecific clinical signs and symptoms and a variable clinical course. Clinical manifestations include fever, malaise, myalgia, meningism, and conjunctivitis, as well as anorexia, abdominal pain, nausea, and vomiting (*1*–*3*,*7*). Initial signs of serious infection can include jaundice, hemorrhage, and hepatosplenomegaly. The most severe form of leptospirosis can occur as Weil syndrome with hepatic and renal failure or massive pulmonary hemorrhage; both forms can progress rapidly to death if untreated (*1*–*3*,*9*).

Leptospirosis is thought to be the most widespread zoonotic disease in the world (*10*). Most reported cases occur in men, most likely due to greater occupational exposure (*11*). Recent outbreaks associated with water sports and recreation include 68 of 304 athletes participating in a 2-week adventure sport race, the Eco-Challenge-Sabah 2000, in Borneo, Malaysia (*12*), and 74 of 639 triathletes in 1998 races in Wisconsin and Illinois, in the largest known outbreak of leptospirosis in the United States (*13*).

## Leptospirosis in the United States

The reported incidence of leptospirosis is 100–200 cases per year in the United States (*2*,*14*), with most (50–100 cases) occurring outside the continental United States in Hawaii (*15*). Leptospirosis is likely underdiagnosed in the United States, with reported incidence depending largely upon clinical index of suspicion (*3*,*6*). Historically, workers in direct contact with animal reservoirs—especially cattle and pig farmers, slaughterhouse workers, veterinarians, and dairy farmers—have been considered to be at increased risk. Recreational exposure may occur from swimming or boating in freshwater lakes formed by runoff or damming (*1*). Infections have been reported in HIV patients (*16*) as well as children (*17*), and urban residential exposure is on the rise, most notably in crowded inner city locations with rat infestations (*5*). Sporadic outbreaks of leptospirosis in the continental United States have occurred in the East, Midwest, and in Texas in the last decade (*5*,*13*,*18*)

## Leptospirosis in California

Leptospirosis has been a reportable disease in California since 1922, even after its removal from the national reportable disease list in 1995, and completion of a case history report form has been required since 1966 (*19*,*20*). In the 20-year period 1982 to 2001, 61 cases of leptospirosis were reported to the California Department of Health Services, for an average of 2.8 cases per year: most of these were imported by vacationers to Hawaii, Malaysia, and other tropical locales outside the state. However, more than half of these cases occurred in the most recent 5 years alone; in the 5-year period 1997–2001, 34 cases were reported, for an average of 6.8 cases of leptospirosis per year (*21*). Thus, the overall incidence of leptospirosis in California appears to be on the rise.

In-state acquisition of leptospirosis in California shows a similar trend. In the 20-year period 1982–2001, 23 reported cases of California leptospirosis were also acquired within California, for an average of 1.15 in-state cases per year. Twelve of these cases occurred in the most recent 5 years, for an average of 2.4 in-state cases per year (*21*). During this period, the population of California rose from 24 million to an estimated 34 million (*22*).

Several trends in the epidemiology of leptospirosis in California are apparent over the last 20 years. Most reported cases appeared in previously healthy adult white men after a recreational exposure to contaminated freshwater. Of the 61 California cases in the last 20 years, at least 77% occurred in men. At least 67% occurred in adults of ages of 20 to 40 years, and at least 70% occurred in persons identifying their ethnicity as white. Recreational exposures accounted for 59% of cases, 16% were occupational, 10% were pet-related, and 15% were due to unidentified exposures. Recreational exposures may be on the rise, as they accounted for a full 85% of exposures in the 34 cases in the most recent 5 years (Table 1) (*21*).

**Table 1:** Features of leptospirosis patients reported in California, 1982–2001

Exposure type	% (n=61)
Recreational	59
Occupational	16
Pet-related	10
Unknown	15
Sex	
Male	77
Female	15
Unknown	8
Race	
White	70
Hispanic	8
Black	2
Unknown	20
Age	
0–20	10
21–40	67
41–60	13
60+	2
Unknown	8

**Figure 1:**
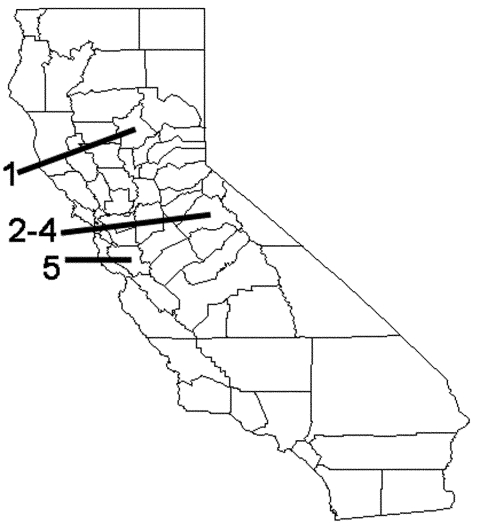
California state map showing case report exposures by county: Case 1, Butte County; cases 2–4, Tuolomne County; case 5, Santa Clara County.

These patterns are also illustrated by the five cases of leptospirosis acquired within the state in the past 3 years (Figure 1). In 1999, there was one sporadic case associated with exposure to freshwater while the patient was duck hunting in Butte County, or possibly following contact with rodent urine in an infested trailer. In 2000, three cases were associated with an outbreak among San Mateo County residents who swam in a reservoir in Tuolomne County while on a houseboat vacation. In 2001, one sporadic case was reported in a woman from Santa Clara County associated with swimming in a muddy pond. We describe these five cases, including the first documented common-source outbreak of leptospirosis acquired in California.

## Diagnosis of Leptospirosis

The diagnosis of leptospirosis can be made by use of rapid serologic assays. The microagglutination test (MAT) has been the reference method for serologic diagnosis of leptospirosis. MAT utilizes antigens from serovars representative of all serogroups, since cross-reactivity between serovars occurs frequently. Though a titer of >1:400 may be considered confirmatory in the appropriate clinical context, a fourfold or greater rise in titer over any time period confirms the diagnosis. A second test is the indirect hemagluttination assay (IHA) for both immunoglobulin (Ig) M and IgG antibodies. A third assay is an enzyme-linked dot immunoassay for IgM antibodies in serum (IgM-Enzyme-Linked ImmunoSorbent Assay [ELISA], the Dip-S-Ticks [Integrated Diagnostics, Inc., Baltimore, Maryland), that appears to have a greater sensitivity early in infection than the other available assays. The IgM-ELISA is more readily available and is less labor-intensive than MAT, especially when paired samples for serologic testing are unavailable. However, the result of IgM-ELISA should be considered preliminary and further confirmation by MAT is recommended. In addition to the above serologic assays, polymerase chain reaction and immunohistochemical (IHC) assays are sensitive microscopic methods of diagnosis if tissue sample is available for testing (*3*,*6*).

## Case Reports

### 1999: Case 1

In mid-January, a 38-year-old man went duck hunting in flooded rice fields in Butte County; he also stayed in a trailer that had stood vacant for some time, where he recalled cleaning up rodent droppings. On January 27, jaundice, pulmonary infiltrates, and renal failure developed. Laboratory studies showed bilirubin of 48 mg/dL and platelets of 13,000/μL.

Serologic testing for hantavirus was negative. IHA for leptospirosis at a private laboratory 9 days later yielded an initially ambiguous titer of 1:50, and a second test 11 days after that yielded a titer of 1:800 (Table 2), greater than the fourfold rise required to confirm the diagnosis. He was treated successfully with doxycycline.

**Table 2:** Acute- and convalescent-phase serologic and microscopy diagnosis of leptospirosis by MAT,^a^ IgM-ELISA, IHA, and IHC assay in California case reports^b^

Case	Case 1		Case 2		Case 3		Case 4		Case 5		
Days post exposure	9	11	15	32	21	36	23	44	Acute	conv	
MAT	ND	ND	Neg	1:3200	Neg	1:800	1:800	1:200	Neg	ND	
ELISA	ND	ND	Neg	Pos	Pos	ND	Pos	ND	Pos	ND	
IHA	1:50	1:800	ND	ND	ND	ND	ND	ND	ND	1:200	
IHC assay	NA		NA		NA		Pos		Pos		

^a^MAT, microagglutination test. Highest MAT titer reported to any leptospira serogroup. ^b^IgM-ELISA, immunoglobulin M–Enzyme-Linked ImmunoSorbent Assay; IHA, indirect hemagluttination assay; IHC, immunohistochemical; NA: not available; ND: not done.

### 2000: Cases 2–4

In 2000, the largest known common-source outbreak of leptospirosis in California involved three men vacationing on a houseboat in the New Melones Lake in Tuolomne County May 5–7. Three of eight men who shared the houseboat reported swimming to a remote cove and hiking along a creek draining into the main reservoir on May 7. They were exposed there to muddied waters after an overnight thundershower. Each of these three men went individually to his own doctor and independent health system in Redwood City 10–15 days later, with a constellation of signs and symptoms including fever, headache, myalgias, nausea, and vomiting. In each, the salient common clinical feature of renal failure developed, manifested by elevated creatinine levels that in one case required hemodialysis.

#### Case 2

A 35-year-old Caucasian man went to an urgent care clinic twice before being admitted to the hospital with fever as high as 39.4°C, myalgias, and renal insufficiency later. Additional symptoms included headache, photophobia, nausea, and anorexia, starting 10 days after returning from the boating trip. Signs included subtle diffuse flushing and conjunctival suffusions. Initial laboratory values showed a mild hepatitis with a rising creatinine that increased daily to peak at 4.0 mg/dL. Liver enzymes were elevated with peak values of alanine aminotransferase (ALT) 258 U/L, alkaline phosphatase 189 U/L, and bilirubin 3.5 mg/dL. Results of examination of the cerebrospinal fluid were normal. Leukocyte count was within normal range at 4.7 k/μL. By the third day of hospitalization, after IV rehydration and broad-spectrum antimicrobial agents, the patient’s fever subsided with improvement of his myalgias and headache. He was discharged and given doxycyline 100 mg twice daily for 10 days. He had no clinical sequelae.

Serologic tests for infectious hepatitis A, B, C, and brucella, and a Monospot test were negative. MAT and IgM-ELISA for leptospirosis were processed at the Centers for Disease Control and Prevention (CDC), and the California Department of Health Services (CDHS), respectively. Titers from serum drawn 15 days after the initial exposure were negative; however, convalescent-phase serum drawn 17 days later tested positive for leptospira by IgM-ELISA at CDHS, and MAT at CDC showed the strongest reaction with serovars from *L. interrogans* serogroups Australis and Mini (Table 2).

#### Case 3

A 35-year-old Caucasian man went to a hospital emergency department 15 days after returning from a house-boating trip to New Melones Reservoir in the Sierra Nevada foothills of northern California. He reported a 4-day history of headache, fatigue, low back pain, myalgia especially of the calves, vomiting, and fever as high as 40.6°C. Signs included erythema of the left flank, with scattered tiny red macules on the legs. Initial laboratory studies showed proteinuria, a creatinine level that peaked at 1.7 mg/dL, and mild transaminasemia, with ALT 182 U/L and alkaline phosphatase 370 U/L. His leukocyte count was within normal range at 9.0 K/μL.

Other serologic tests, including for hepatitis A, B, and C, rickettsia, brucella, and ehrlichia, were all negative. He took doxycycline 100 mg orally twice daily for 10 days and made a complete recovery. MAT and IgM-ELISA for leptospirosis were processed at CDC; titers from serum taken 21 days and 36 days after the houseboat trip were most strongly positive with serovars from *L. interrogans* serogroup Mini (Table 2).

#### Case 4

A 38-year-old man went to the hospital emergency department 13 days after the houseboat excursion, with fever of 37.8°C, tea-colored urine, myalgias, chills, headache, anorexia, nausea, and abdominal pain. He was admitted to the hospital with oliguric acute renal failure. Peak elevated laboratory values included a creatinine of 17.3 mg/dL, blood urea nitrogen 82 mg/dL, urobilinogen 4+ EU/dL, and urine protein 100 mg/dL. His leukocyte count was within normal range at 5.6 k/μL.

**Figure 2:**
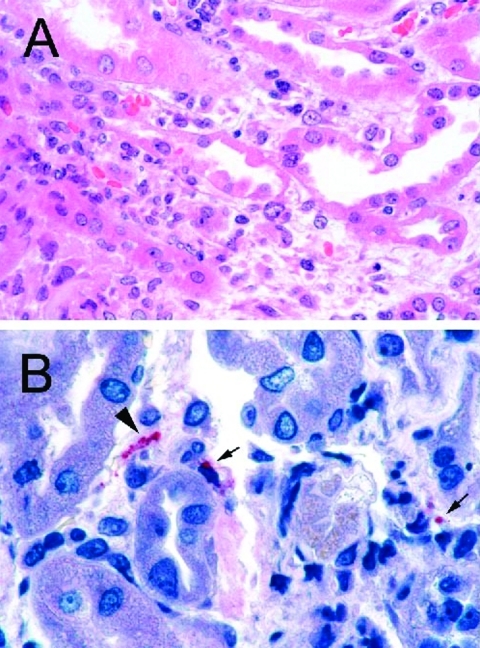
A: Renal biopsy shows inflammatory cell infiltrate in the interstitium and focal denudation of tubular epithelial cells. Hematoxylin and Eosin; original magnifications x100. B: Immunostaining of fragmented leptospire (arrowhead) and granular form of bacterial antigens (arrows). Original magnifications x158.

Serologic tests for hepatitis A, B, C, rickettsiae, and ehrlichiae were negative. Renal biopsy 17 days post exposure showed acute tubulointerstitial nephritis (Figure 2A), which is reported to be the most characteristic renal lesion observed in leptospirosis (*23*,*24*). Ultrasonography showed bilateral pleural effusions and echogenic kidneys. He received broad-spectrum antimicrobials and dialysis and remained hospitalized for almost a month. His acute renal failure resolved, and he made a full recovery. MAT and IgM-ELISA results for leptospirosis were processed at CDC; titers from serum taken 23 days and 44 days postexposure were most strongly positive with serovars from *L. interrogans* serogroup Mini (Table 2). Immunohistochemical assay for leptospira was performed at CDC and showed granular immunostaining of leptospira in the kidney biopsy.

### 2001 Case 5

A 53-year-old woman went hiking in the foothills of Mount Hamilton in Santa Clara County on September 1, 2001, where she swam in a muddy pond. Eleven days later, she had the onset of shaking chills, fever of 38.9°C myalgias, ankle and wrist pain, and headache. Her fever resolved, but 4 days later nausea, vomiting, diarrhea, and a worsening headache developed and she went to urgent care. Initial laboratory test results including cerebrospinal fluid, blood urea nitrogen, and creatinine were normal, and she was treated for gastroenteritis with promethazine HC1 and fluids.

Three days later, oliguria developed. On September 19, she was admitted with oliguric renal failure and signs including an erythematous macular rash on her face and pretibial macular blanching lesions on her legs. Over the next few days, a persistent cough with rales developed, and peak levels of blood urea nitrogen were 101 and creatinine were 9.5. Urinalysis showed 1+ blood, 6–10 erythrocytes, 0–3 leukocytes, and 3+ protein. Though her leukocytes were normal at 5.9 k/μL with 84% polymorphonuclear leukocytes (PMNs), her platelet count was low at 122,000. Mild transaminasemia also developed as well as an elevated International Normalized Ratio (INR). She was treated with high-dose penicillin G and corticosteroids.

A renal biopsy, which showed tubulointerstitial inflammation, was performed. Immunohistochemical assays for several organisms were performed at CDC; the results were negative for hantavirus and spotted fever group rickettsiae, while immunohistochemical assay for leptospira showed granular immunostaining in the renal biopsy to confirm the diagnosis of leptospirosis (Figure 2B). The patient was discharged after 6 days on doxycycline 100 mg twice daily for 10 days and made a full recovery. Acute-phase serum was positive for leptospira IgM antibody by ELISA but negative for all serovars by microagglutination test at CDC. Convalescent-phase serum was positive for Leptospira by indirect hemagluttination assay at a private laboratory (Table 2).

## Environmental Investigation

The treating physicians in the 2000 New Melones outbreak initiated a public health inquiry to investigate potential environmental risks. This investigation identified potential sources of infection but did not identify a definitive source.

Five weeks after the New Melones exposures, a multidisciplinary team traveled to the reservoir to sample environmental sources for leptospires and to direct potential public health interventions. The physician for the first case, the state public health veterinarian, environmental health researchers from the University of California at Berkeley, and the local environmental health and sheriff’s departments collaborated to better define the origins of this disease outbreak.

The team documented cattle herds grazing in the region above Wolf Gulch, where the three patients were presumed to have been infected. Leptospirosis is commonly transmitted following flooding that creates standing water in regions where cows or other animals graze (*1*–*3*,*25*). Though wildlife, including rodents, ungulates, and small carnivores, is common in this area, the proximity of the cattle and evidence that leptospirosis vaccination does not prevent shedding by cattle implicate these herds in the transmission of infection. However, the cattle were not tested for leptospiral antibodies because of their likely vaccination history. Furthermore, New Melones is a lake created by a dam of the Stanislaus River, and runoff between April and July is usually from snowmelt. After a warm, dry period of almost 3 weeks with temperature maximums in the high 70s and 80s, rain fell on May 6 and 7 totaling less than 1/2 inch (*26*). This weather shift could have provided the mechanism for contaminated animal urine to drain into the creeks surrounding the reservoir.

Using the Moore swab technique for water collection, the team collected 5 gallons of water from three sites in the reservoir, filtering them through sterile cotton plugs which were then incubated in leptospiral media (Ellinghausen McCullough Johnson Harris [EMJH] medium) at 29°C for several days. Though two of the three samples were positive for spirochetes upon darkfield microscopy, they were negative by polymerase chain reaction using primers specific for pathogenic strains of leptospira, a result of ambiguous significance in this case.

Once the working diagnosis of leptospirosis was formulated, the county public health departments were notified where the cases were reported (San Mateo County) and where the exposures occurred (Tuolomne County). Public health notices warned primary caregivers and emergency departments in these areas to consider leptospirosis in patients with fever, headache, and myalgia who had been exposed to the water of Lake Melones. The Tuolomne General Hospital emergency department identified a potential fourth case of leptospirosis on June 6, in a 23-year-old man with headache, myalgias, chills, neck pain, and vomiting, 5 days after swimming in New Melones Lake; symptoms resolved with doxycycline, and leptospirosis titers were not obtained.

## Common Clinical Features

Salient features in the California cases include high fever with uniform renal impairment and mild hepatitis. Further clinical features shared in all five cases of leptospirosis in California include headache, nausea/vomiting, and myalgia. All five patients had elevated creatinine levels and normal leukocyte counts. All patients were treated with doxycycline and made complete recoveries after variable levels of severity in the course of their illnesses.

## Discussion

Leptospirosis is a reemerging infection in California, with most cases appearing in young adult white men after recreational freshwater exposures. This report includes the first documented common-source outbreak of human leptospirosis in California.

In addition to leptospirosis, the differential diagnosis for a patient with fever, fatigue, stiff neck, headache, nausea and vomiting, and myalgias should include mononucleosis, hepatitis viruses B and C, meningitis, and zoonotic infections such as brucellosis, tularemia, hantavirus, dengue, Colorado tick fever, plague, rickettsiosis, ehrlichiosis, and Q fever. With a history of rural freshwater exposure, additional infections worth considering include hepatitis A, *Salmonellosis* spp., toxoplasmosis, and *Naegleria* meningitis. The differential diagnosis may be expanded with other manifestations, such as pulmonary involvement, thrombocytopenia, abdominal pain, or other signs. Laboratory values to consider include complete blood count, urinalysis, and liver function tests including creatinine. Lumbar puncture may be indicated. Leptospirosis has been considered easily treatable with penicillin or doxycycline, though clear evidence-based practice guidelines are still lacking (*27*,*28*).

The diagnosis of leptospirosis may be complicated by the difficulty of culturing the spirochetes from the urine of human patients. There is a biohazard concern for laboratory workers, and MAT is becoming progressively less available nationwide. Immunohistochemical assay is a sensitive diagnostic method when tissue sample is available for testing; it can demonstrate the presence of leptospiral antigens with a morphologic context for clinico-pathologic correlation. Furthermore, there is no biosafety issue involved in the diagnostic procedure, and the test can be performed on archival pathologic specimens. More sensitive serologic assays, such as polymerase chain reaction, ought to aid diagnosis as they are becoming more readily available. Though serovar identification might implicate a particular animal reservoir for an outbreak, cross-reaction between serovars may further obscure the definitive source of disease.

Freshwater recreational exposures are the major route of exposure for recent cases acquired in California. Recreation, either domestic or abroad, was the means of exposure by which 59% of California residents acquired leptospirosis in the 20-year period of 1982 to 2001, increasing to 85% in the most recent 5 years. Fresh water may be contaminated with the urine of infected animal reservoirs such as rodents, wild ungulates, and livestock.

Limitations of environmental studies include the lengthy time lapse between exposure to onset, diagnosis, and case investigation, during which time the microbe may be washed away from an aquatic environment. Regions of environmental perturbation including dams or recently expanded recreational areas may be increasing sources of contact with leptospires. Transmission appears to follow warm weather with flooding rains.

Since leptospirosis is a reemerging zoonotic disease, and its presence in animals is widespread, a high index of suspicion for this treatable illness is needed. Because leptospirosis may progress rapidly with severe sequelae, this reemerging infection deserves consideration in febrile patients with a history of freshwater recreation, even in states with a low reported incidence of infection.
